# Effects of Narrow Band UVB (311 nm) Irradiation on Epidermal Cells

**DOI:** 10.3390/ijms14048456

**Published:** 2013-04-17

**Authors:** Adam Reich, Karolina Mędrek

**Affiliations:** Department of Dermatology, Venereology and Allergology, Wroclaw Medical University, Chałubińskiego 1, Wrocław 50-368, Poland; E-Mail: karolinamedrek@op.pl

**Keywords:** cell death, epidermis, keratinocytes, NB-UVB, phototherapy

## Abstract

Ultraviolet radiation (UVR) is known to be one of the most important environmental hazards acting on the skin. It was revealed that chronic exposure to UVR accelerates skin aging, induces immunosuppression and may lead to the development of skin cancers. On the other hand, UVR has been shown to be effective in the treatment of numerous skin diseases and thus, various phototherapy modalities have been developed to date. Narrow-band ultraviolet B (NB-UVB) emitting a light with a peak around 311 nm has been demonstrated to be effective in the treatment of various skin disorders; currently it is one of the most commonly used phototherapy devices. Despite NB-UVB has been developed more than 30 years ago, the exact mechanism of its therapeutic action remains poorly understood. To date, most of NB-UVB effects were attributed to its influence on immune cells; however, nearly 90% of NB-UVB irradiation is absorbed by epidermis and keratinocytes seem to be important players in mediating NB-UVB biological activity. Here, we have reviewed the current data about the influence of NB-UVB on epidermal cells, with a special emphasis on cell proliferation and death.

## 1. Introduction

Ultraviolet radiation (UVR) is known to be one of the most important environmental hazards acting on the skin. It was revealed that chronic exposure to UVR accelerates skin aging, induces local and systemic immunosuppression and may lead to the development of melanoma and non-melanoma skin cancers [1,2]. Immunomodulatory properties of UVR are initiated via a cascade of events beginning with the absorption of UVR energy by endogenous chromophores, including nuclear DNA, trans-urocanic acid, and components of cell membranes [3]. Due to highly energetic photons, UVB more efficiently than UVA induces changes in the skin causing a carcinogenic DNA damage and various epigenetic effects [2,4]. DNA mutations after UVB exposure include single-stranded DNA breaks and intra-stranded DNA base-crosslinkings via formation of cyclobutane pyrimidine dimers and 6-4 pyrimidine-pyrimidone photoproducts [5,6]. Moreover, UVB, as with UVA, is able to induce cell damage due to generation of reactive oxygen species.

To minimize the negative influence of UVR human skin possesses a number of protective mechanisms including cell-cycle arrest, DNA repair and programmed cell death for situation, when DNA damage cannot be repaired [4,7–14]. Failure of any of these mechanisms may lead to carcinogenesis and development of skin cancers [8].

Despite the above mentioned hazardous properties, UVR was shown to be effective in the treatment of numerous skin diseases. In early 1980s, it was observed that the beneficial effect of UVB is mainly related to wavelengths between 295 and 313 nm, and a range between 310 and 313 nm seemed to have the best safety profile [9,10]. Consequently a lamp, emitting UVB irradiation with a peak at 311–312 nm (known as narrow-band ultraviolet B—NB-UVB) and excluding shorter, photobiologically more dangerous wavelengths, has been developed. NB-UVB was demonstrated to be effective in the treatment of such skin disorders, like plaque psoriasis, primary cutaneous T-cell lymphomas, atopic eczema, seborrheic dermatitis, pityriasis rubra pilaris, lichen planus, prurigo nodularis, uremic pruritus or even vitiligo [11,12]. Since its development, use of NB-UVB has been prompted by a combination of its therapeutic efficacy and good safety profile regarding acute adverse events. However, concerns with regard to long lasting consequences still remains unsolved [13,14].

Remarkably, the exact mechanism of NB-UVB therapeutic action remains poorly understood; it is even unclear, whether local or systemic effect is more important [12,15–18]. To date most of NB-UVB action has been attributed to its influence on immune cells, however, nearly 90% of NB-UVB irradiation is absorbed by the epidermis, and it seems probable, that keratinocytes, a major cellular component of epidermis, can also be, at least partially, affected by NB-UVB and may partake in mediation of therapeutic efficacy of this phototherapy. Here, we have reviewed current data about the influence of NB-UVB on epidermal cells, with a special emphasis on cell proliferation and death.

## 2. Narrow Band UVB (311 nm) and Keratinocyte Proliferation

Several studies have shown that NB-UVB inhibits proliferation and is able to induce apoptosis in human keratinocytes, both *in vitro* and *in vivo*[19–25]. Luo *et al.*[21,23] observed, that single low dose of NB-UVB irradiation (dose below or equal to 100 mJ/cm^2^) in combination with retinoids (acitretin or tazarotene) inhibits the cell growth *in vitro* to significantly greater extent than therapy with these drugs alone. As suggested by the authors [21,23] this synergistic effect might be, at least partially, mediated by heparin-binding epidermal-growth-factor like growth factor (HB-EGF), which was up-regulated both upon NB-UVB irradiation and retinoid treatment [21]. Unlike other members of the family of epidermal growth factors, HB-EGF slows proliferation of keratinocyte and, on the other hand, accelerates their migration, a feature that might be important in wound healing [26]. Inhibition of cell growth during repeated treatment with NB-UVB may also be linked with GATA3-mediated pathways [27]. GATA3 is a transcription factor with two zinc finger motifs which binds to a six-nucleotide sequence (A/T)GATA(A/G) [28]. It is essential for correct formation of the epidermal barrier, regulation of epidermal differentiation and desquamation [29,30]. The latter effect is mediated via activation of kallikrein 1 [30]. GATA3 has been shown to be downregulated in patients with psoriasis, a chronic inflammatory skin disease characterized by a high keratinocyte proliferation rate [27]. Standard NB-UVB therapy increased the expression of GATA3 mRNA in psoriatic skin towards the levels found in healthy controls and the restoration of GATA3 expression correlated with psoriasis improvement [27]. Furthermore, histological examination showed decreased epidermal thickness, improved epidermal differentiation and significantly reduced number of proliferating cells in NB-UVB-treated mouse skin with psoriasiform dermatitis as compared to the skin of control animals [27]. GATA3 downregulation was associated with decreased expression of other transcription factors, like the psoriasis susceptibility gene TNFAIP3 and the Notch ligand jagged 2 (both acting as negative regulators of inflammation), and genes regulating epidermal differentiation, such as the transcription factor AP2-α (TFAP2A) and apoptosis-inducing FAS ligand. In general, low GATA3 expression in human psoriatic and GATA3-deficient murine epidermis coincided with altered expression of genes involved in cell differentiation, proliferation and apoptosis [27]. Importantly, treatment with NB-UVB restored these alterations and led to improvement of psoriatic lesions, both clinically and histologically [27]. In addition, repeated NB-UVB exposures decreased the level of survivin (an anti-apoptotic protein) in psoriatic epidermis *in vivo*, enabling epidermal cells to undergo apoptosis [31].

Regarding single NB-UVB irradiation, inhibition of epidermal cell growth *in vivo* was observed after the dose equal or higher than at least 200 mJ/cm^2^[21,22]. It seems, that higher doses of NB-UVB induces DNA damage leading to cell-cycle arrest, which gives cells time to repair DNA mutations. Usually the inhibition of keratinocyte proliferation after single NB-UVB dose lasted less than 48 h [22]. However, if the dose was higher than 400 mJ/cm^2^, apoptosis of keratinocytes (HaCaT cell line) was observed.

## 3. Narrow Band UVB (311 nm) and Apoptosis of Keratinocytes

NB-UVB is able to evoke apoptosis in epidermal cells in a similar pattern as the broad-band UVB does. However, the required dose to induce the same amount of apoptosis and cell death as in the broad-band UVB treated cells is about five to ten times higher [19]. As shown by Schindl *et al.*[19], using human squamous cell carcinoma-derived cell line a dose of 2048 mJ/cm^2^ of NB-UVB was required to obtain 50%–60% of cells positive for propidium iodide, which identifies cells with the loss of membrane integrity that is characteristic feature of later phases of cell death. However, a significant degree of apoptosis was already observed at the doses of about 800–1000 mJ/cm^2^ depending on studied cell types and conditions of irradiation [20,22]. Importantly, an apoptotic dose of NB-UVB is very similar to the minimal erythema dose (MED) of NB-UVB observed by Gloor and Scherotzke [32] in Caucasian subjects, indicating that indeed UV-induced erythema might be an inflammatory response to the appearance of “sunburn cells” (*i.e*., apoptotic cells) in native human epidermis. Broad-band UVB and NB-UVB at equivalent MED doses were equally potent in inducing p53 and p16 expression as well as apoptosis in healthy volunteers [33]. As shown by Aufiero *et al.*[20] caspase 3 activation was a direct response to NB-UVB mediated apoptosis *in vitro*. A recent study by Weatherhead *et al.*[34] nicely documented that apoptosis of keratinocytes is a major hallmark of therapeutic activity of NB-UVB in psoriasis. It was observed that irradiation with NB-UVB but not with equally erythemogenic doses of 290 nm significantly increased apoptosis of lesional epidermal cells and the number of apoptotic cells peaked at 16–24 h [34]. They were located in the basal and suprabasal epidermis. Furthermore, it was demonstrated, that apoptosis occurs also *in vivo* during routine clinical therapy with NB-UVB and a computational model of psoriatic epidermis revealed that induction of keratinocyte apoptosis (but not inhibition of keratinocyte proliferation) in psoriatic lesions is sufficient for explanation of clearance of psoriatic plaques within the timeframes observed clinically during NB-UVB therapy [34,35]. Based on this study it seems, that, indeed, keratinocytes are the major target of NB-UVB therapeutic efficacy.

**Figure 1:**
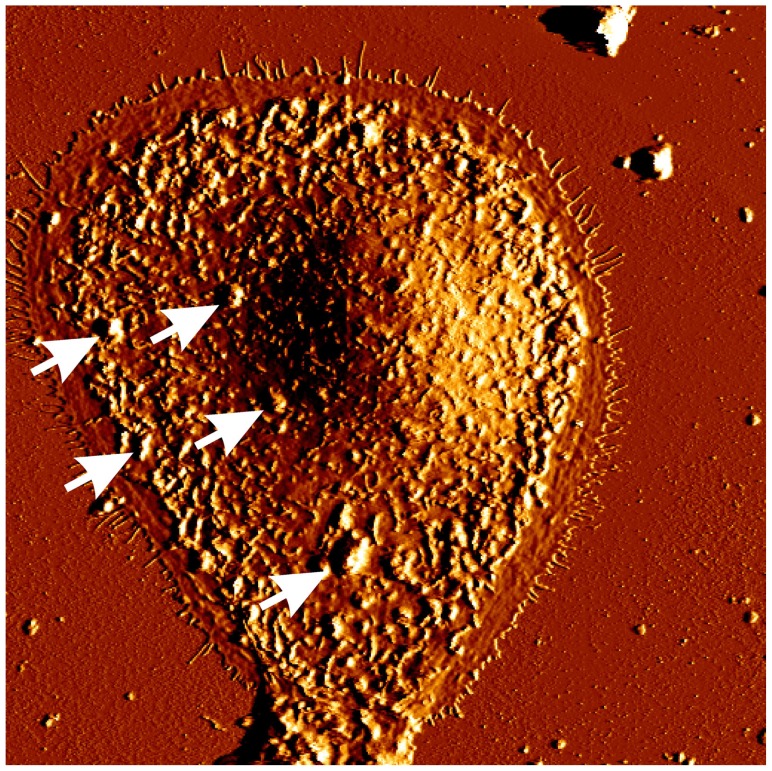
Morphology of a single keratinocyte 48 h after irradiation with 800 mJ/cm^2^ of narrow-band UVB showing numerous bleb-like protrusions on the cell surface (arrows) (AFM micrograph, retrace deflection image, image size: 85 × 85 μm)

Irradiation with apoptosis-inducing NB-UVB doses strongly altered cultured keratinocyte morphology, both on the cell surfaces as well as intracellulary. NB-UVB irradiation led to disintegration of nuclear and cell membranes as well as to severe cytoplasmic vacuolization [20,22]. Using atomic force microscopy (AFM) which enables to study cell morphology at a high resolution in the physiologically relevant, aqueous environment it was shown that one of the most prominent morphological alterations on surfaces of NB-UVB irradiated cells was the formation of irregularly distributed, bleb-like protrusions (Figure 1) [22]. Similar structures were observed by other authors on the keratinocytes irradiated with broad band UVB, but not with UVA, and were found to contain nuclear ribonucleoproteins [36–39]. They were also shown to contain nuclear antigens recognized by antinuclear antibodies like Ro/SS-A or La/SS-B from patients suffering from lupus erythematosus, a common autoimmune multi-organ disease [24,39]. These observations further supported the damage of cell nucleus by NB-UVB irradiation and underlined a possible explanation of photosensitivity in patients with autoimmune connective tissue disorders having antinuclear antibodies. NB-UVB irradiation also provoked significant rearrangement of the cytoskeleton, causing thinning of microfilaments and their redistribution to the cell periphery [22].

Disruption of cellular membranes of cultured cells upon NB-UVB irradiation was accompanied by significant changes in the cellular lipid content [25]. Irradiation with NB-UVB resulted in the increased production of ceramides—*i.e.*, lipid species which are considered to be signal transducers of a variety of cell stressors, including reactive oxygen species, cytokines, exposure to chemotherapeutic agents, irradiation or exogenous lipopolysaccharides [40]. Increased ceramide level was able to provoked cell cycle arrest and/or apoptosis in a variety of cell types [40], suggesting that overproduction of ceramides after NB-UVB exposure may be another mechanism of apoptosis induction in heavily irradiated cells. Interestingly, the content of some PC- and PE-ethers (1-alkyl,2-acylglycerophosphocholines and 1-alkyl,2-acylglycerophosphoethanolamines) also increased in irradiated cells [25]. Although their role in response to irradiation remains unclear, it is likely that they were also associated with ongoing apoptosis, as they changed similarly to ceramides. Their highest level was achieved 12 h post irradiation and dropped later down to the baseline concentration. It seems probable that formation of PC- and PE-ethers might be a consequence of lipid peroxidation caused by NB-UVB irradiation and these lipid species might be toxic for epidermal cells as it was previously reported that synthetic ether phospholipids (e.g., 1 *O*-octadecyl-2-Omethyl-rac-glycero-3-phosphocholine) were able to induce apoptosis in a number of cell lines [41,42]. In addition, cells irradiated with 800 mJ/cm^2^ of NB-UVB were found to accumulate triacylglycerols (TAG), which peaked at the later stages of apoptosis [25]. Several previous studies also reported accumulation of TAGs in various cell types upon different apoptotic stimuli [43–48]. This was observed in cells stimulated by free fatty acids and FAS pathway activation. Increased TAG content is a widely recognized apoptosis marker, unrelated to the factors inducing this process. Al-Saffar *et al.*[44] suggested that TAG accumulation in Jurkat T cells might be a result of altered PC metabolism. It was found that PC biosynthesis was inhibited during apoptosis at the level of cytidine diphosphate-choline: 1,2-DAG choline phosphotransferase and that the accumulation of PC substrates activated TAG production [44,49,50]. It was speculated that TAG accumulation in non-adipose cells might constitute a protective mechanism against apoptosis, due to increased ability to incorporate free fatty acids into TAGs, a phenomenon which decreases the apoptosis ratio. In addition, the magnitude of TAG accumulation correlates with cell survival upon exposure to palmitate [45,47]. However, the exact mechanism remains controversial, as other authors suggested that TAG accumulation enhanced ceramide synthesis and reactive oxygen species production, eventually causing cell death [51,52]. In conclusion, NB-UVB altered the lipidome of keratinocytes in a pattern of changes consisting with unfolding apoptosis. It is still unclear, whether these observations have clinical relevance, however, one may speculate that increased synthesis of ceramides and TAGs after NB-UVB irradiation in apoptosis-undergoing cells may help in restoration of abnormal epidermal lipid composition in patients suffering from atopic dermatitis or psoriasis, an idea which has been already suggested by Wefers *et al.*[53]. Despite single NB-UVB irradiation increased ceramide content in epidermis for a short time only, it seems probable that repetitive therapy with NB-UVB, as it is usually applied, may lead to durable increase of ceramides and TAGs in the skin. Therefore, this unwanted effect from the point of view of single cells might be beneficial for patients suffering from certain skin disorders.

## 4. Narrow Band UVB (311 nm) Action on Other Cell Types

NB-UVB is also able to induce apoptosis of skin cells other than keratinocytes. Lymphocytes seems to be more sensitive to NB-UVB irradiation as compared to keratinocytes, as slightly lower doses were required to induce apoptosis of former cells [20]. It was also shown that NB-UVB evoked apoptosis in T lymphocytes infiltrating epidermis more efficiently than broad band UVB [54]. Besides inducing apoptosis, NB-UVB treatment lowered production of proinflammatory cytokines such as IL-1α, IL-2, IL-5 and IL-6, whereas the synthesis of anti-inflammatory IL-10 was significantly augmented [55]. Furthermore, NB-UVB also decreased number of epidermal Langerhans cells from 4 h up to 48 h post irradiation and multiple exposures of NB-UVB reduced the density of Langerhans cells in epidermis by about 20% [33,56]. However, the reduction of the density and function of Langerhans cells in the skin and their migration to the draining lymph node upon NB-UVB irradiation was less pronounced than after broad-band UVB exposure [57]. On the other hand, NB-UVB evoked greater depletion of T lymphocytes in psoriatic plaques than broad-band UVB [58]. Due to the depth of NB-UVB penetration [59], these observations mostly apply for immune cells present in the epidermis and papillary dermis.

NB-UVB also acts on the epidermal pigment cells. NB-UVB remains a gold standard in the treatment of vitiligo, an immune-driven disease with loss of melanocytes [60]. It was shown, that NB-UVB irradiation with a dose of 25 mJ/cm^2^ increased proliferation rate of NCCmelan5 cells, a melanoblast cell line [61]. Interestingly, other study did not show a direct stimulatory effect of NB-UVB on melanocyte growth, a difference that could be explained by different cell line (*i.e*., cultured primary human melanocytes) studied [62]. However, the melanocyte proliferation was stimulated by supernatant of NB-UVB irradiated keratinocytes, an effect that seems to be mediated via endothelin-1 [62]. On the other hand, NB-UVB directly increased the melanocyte migration [62]. NB-UVB was also shown to directly stimulate hair follicle-derived neural crest stem cells to differentiate into melanocyte lineage. NB-UVB enhanced the mobility of NCCmelan5 cells via upregulation of pp125FAK (a protein known to be involved in cell mobility after phosphorylation) as well as increased melanin formation and tyrosinase expression [61,63]. Remarkably, irradiation with higher NB-UVB dose (50 mJ/cm^2^) decreased the viability of NCCmelan5 cells by about 30% [61].

## 5. Conclusions

In summary, NB-UVB is able to inhibit cell proliferation as well as is able to induce apoptosis of various cell types. This action may be responsible for its therapeutic activity in diseases with a high proliferation rate, such as psoriasis. On the other hand, the induction of apoptosis upon NB-UVB irradiation may lead to the surface expression of nuclear antigens, which next may contribute to aggravation of autoimmune connective tissue diseases driven by antinuclear antibodies. Over the last decade, new data have arisen on the mechanism of action of NB-UVB, but still the exact mechanism of its therapeutic efficacy is far to be fully understood. It is e.g., not clear, why some patients with psoriasis respond to NB-UVB therapy, while others do not show any clinical improvement. It is also still not known which cells (keratinocytes, lymphocytes or maybe Langerhans cells) are a primary target of NB-UVB action and whether NB-UVB is able to induce a systemic immunomodulatory effect or acts only within the irradiated skin area. In addition, to date most studies on NB-UVB mechanism concentrated mainly on psoriasis, but these data do not have to reflect the NB-UVB action in other diseases, which are successfully treated with NB-UVB. Therefore, future studies are needed to better understand the biological activity of NB-UVB irradiation.
